# Crucial X-ray Examination in Dentistry: A Case of Hidden Carious Lesion in Tooth #20

**DOI:** 10.7759/cureus.96874

**Published:** 2025-11-14

**Authors:** Liubov Chorna

**Affiliations:** 1 Pediatric Dentistry, Poltavsʹkyy Derzhavnyy Medychnyy Universytet (PDMU), Kyiv, UKR

**Keywords:** dental caries diagnosis, dental x-ray, pediatric dental treatment, preventive maintenance, teeth caries

## Abstract

This clinical case demonstrates how timely and preventive radiographic examination enabled the early detection of a carious lesion in tooth #20, which appeared clinically healthy and asymptomatic. Periapical (PA) radiography identified the lesion before its progression into pulpitis. The patient, a 13-year-old female, reported no complaints and exhibited no visible signs of decay, underscoring the importance of regular diagnostic radiographs. Early diagnosis facilitated conservative restorative treatment, optimizing clinical outcomes and minimizing the risk of complications. This case emphasizes the value of preventive radiographic screening as part of routine dental examinations.

## Introduction

Routine radiographic examination in dentistry is often underestimated by patients, who may refuse it when no symptoms are present. However, the absence of discomfort does not necessarily indicate the absence of disease. Hidden carious lesions can progress silently beneath intact enamel, leading to extensive structural damage and potential pulp involvement before any visible or tactile changes occur [1].

In this case, tooth #20 demonstrated that even clinically sound teeth may conceal moderate decay detectable only through radiographic evaluation. This report emphasizes the crucial role of routine radiographic examination in identifying early pathological changes, enabling timely and minimally invasive interventions, and ultimately preserving both tooth vitality and overall oral health.

## Case presentation

A 13-year-old female patient presented for a routine dental check-up with no specific complaints. Her medical history was unremarkable, and she reported regular dental visits along with good oral hygiene habits. During the clinical examination, tooth #20 appeared clinically intact, showing no discoloration, cavitation, or surface irregularities. The patient reported no pain or sensitivity at the time of examination.

**Figure 1:**
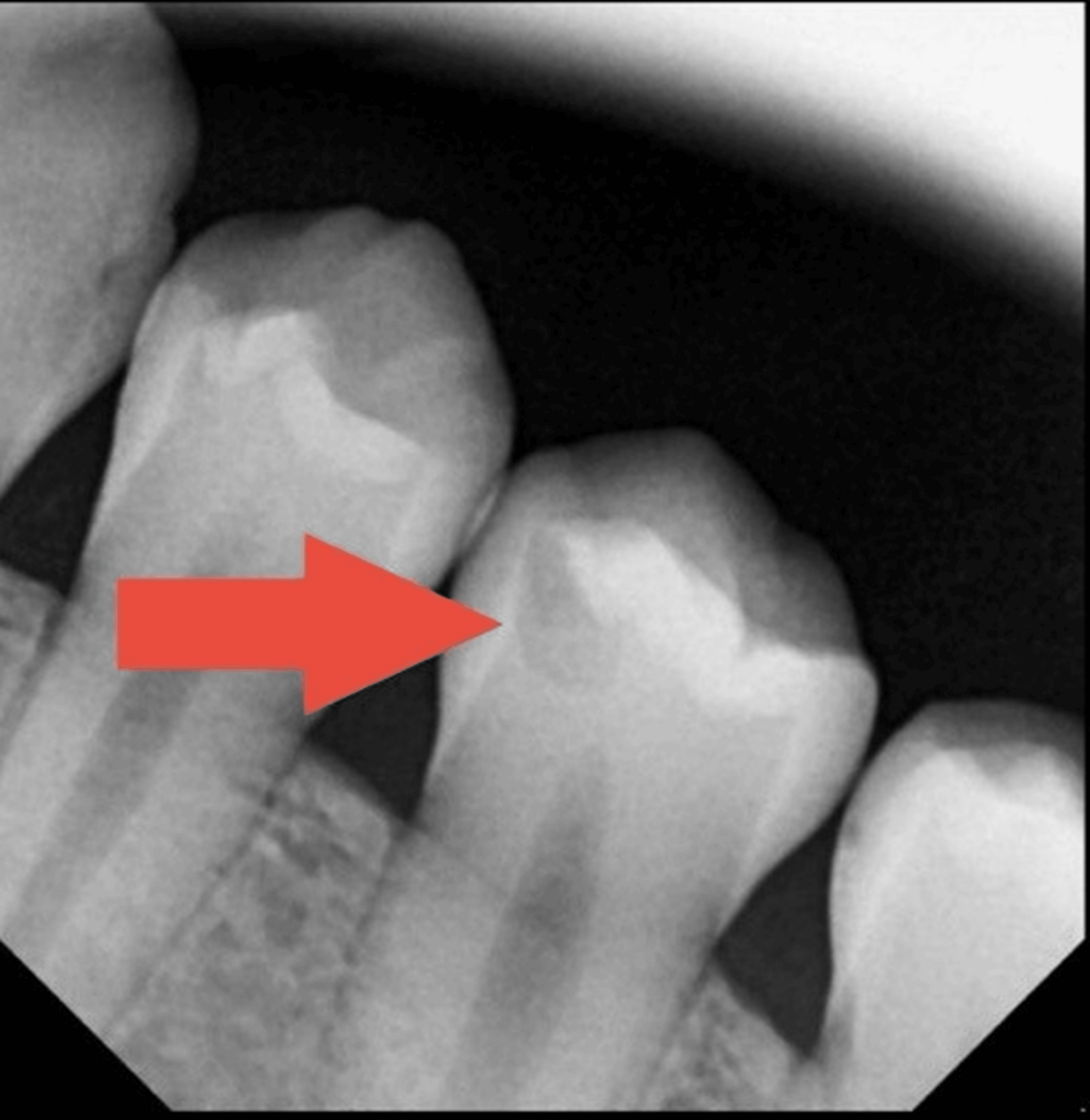
Preoperative periapical radiograph showing a hidden occlusal carious lesion on tooth #20.

As part of a routine radiographic examination, a periapical (PA) radiograph was taken to evaluate the general dental condition. The image (Figure 1) revealed a hidden occlusal carious lesion in tooth #20, which was not visible during clinical inspection. In addition to the radiographic assessment, standard clinical diagnostic tests were performed. Percussion was negative, the cold test was positive within normal limits, and palpation findings were normal. Clinically, the occlusal surface appeared intact, with no visible cavitation or discoloration, consistent with an early visual International Caries Detection and Assessment System (ICDAS) presentation (sound to ICDAS 1-2). However, radiographically, the lesion represented moderate dentinal caries. According to the American Dental Association (ADA) Caries Classification System [2], the radiographic appearance corresponded to a D2 category, with radiolucency extending into the middle third of the dentin without approaching the pulp. Based on these findings, the case was diagnosed as hidden occlusal dentinal caries, and a preventive and conservative restorative approach was selected to preserve tooth vitality. The treatment plan and its benefits were explained in detail to the patient and her guardian, and informed consent was obtained.

**Figure 2:**
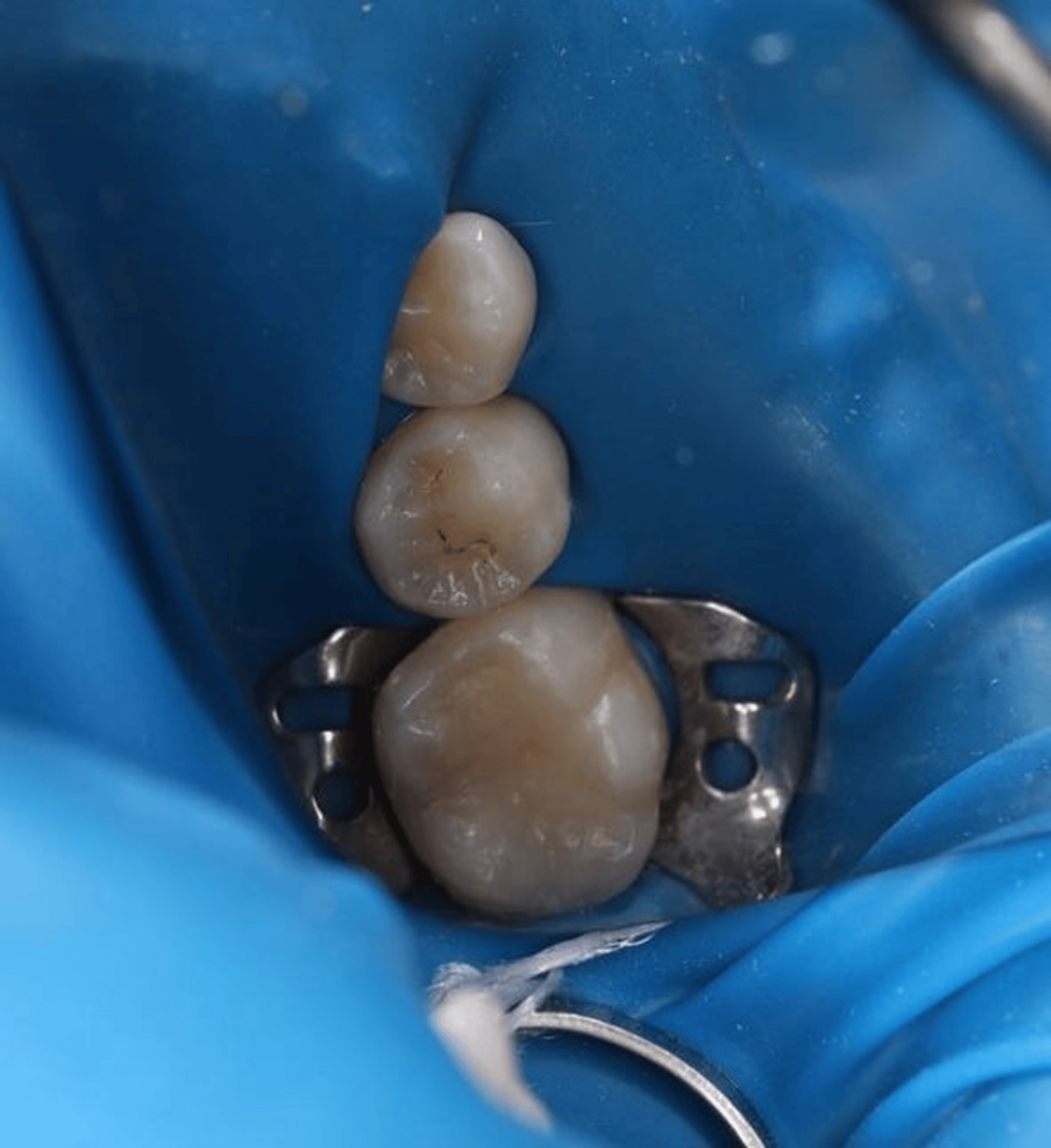
Placement of a rubber dam for tooth isolation before cavity preparation.

Local anesthesia was administered using 1.8 cc of lidocaine 2% with 1:100,000 epinephrine (mandibular block) and 1.8 cc of Septocaine 4% with 1:200,000 epinephrine (buccal and infiltration) [3]. In addition, a small amount of anesthetic was gently deposited into the interdental papilla to ensure complete soft-tissue anesthesia and patient comfort. Once adequate anesthesia was achieved, the tooth surface was cleaned using ProphyFlex air polishing to remove plaque and surface deposits. A rubber dam was then placed to isolate the tooth and maintain a dry operating field (Figure 2).

**Figure 3:**
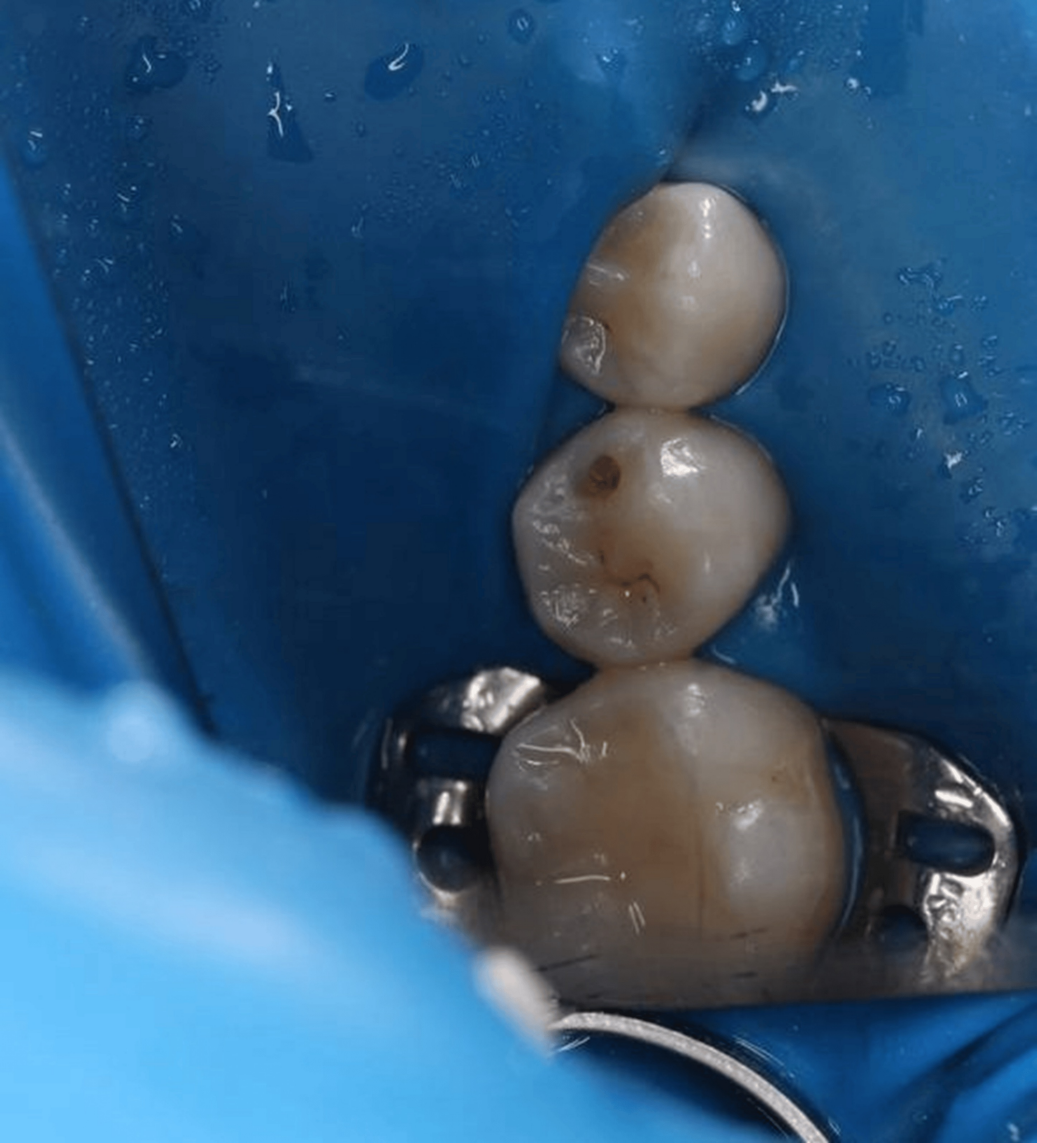
Open carious cavity after initial access preparation showing the extent of dentin involvement.

**Figure 4:**
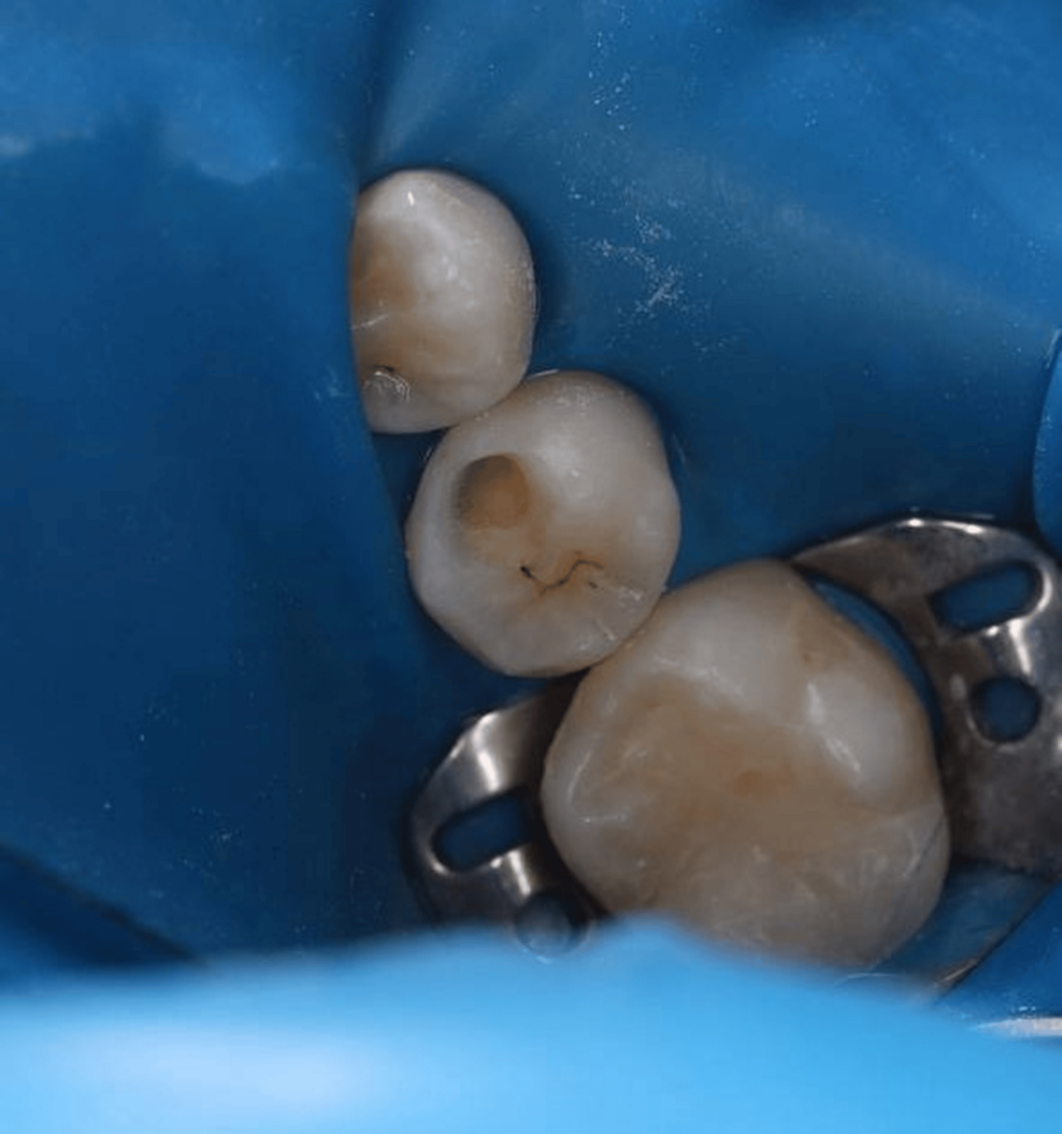
Cavity after complete removal of softened dentin using a low-speed handpiece and spoon excavator.

Access to the carious lesion was obtained using a high-speed handpiece with water spray (Figure 3), and the softened dentin was gently removed with a low-speed handpiece and a spoon excavator, preserving sound tooth structure (Figure 4).

**Figure 5:**
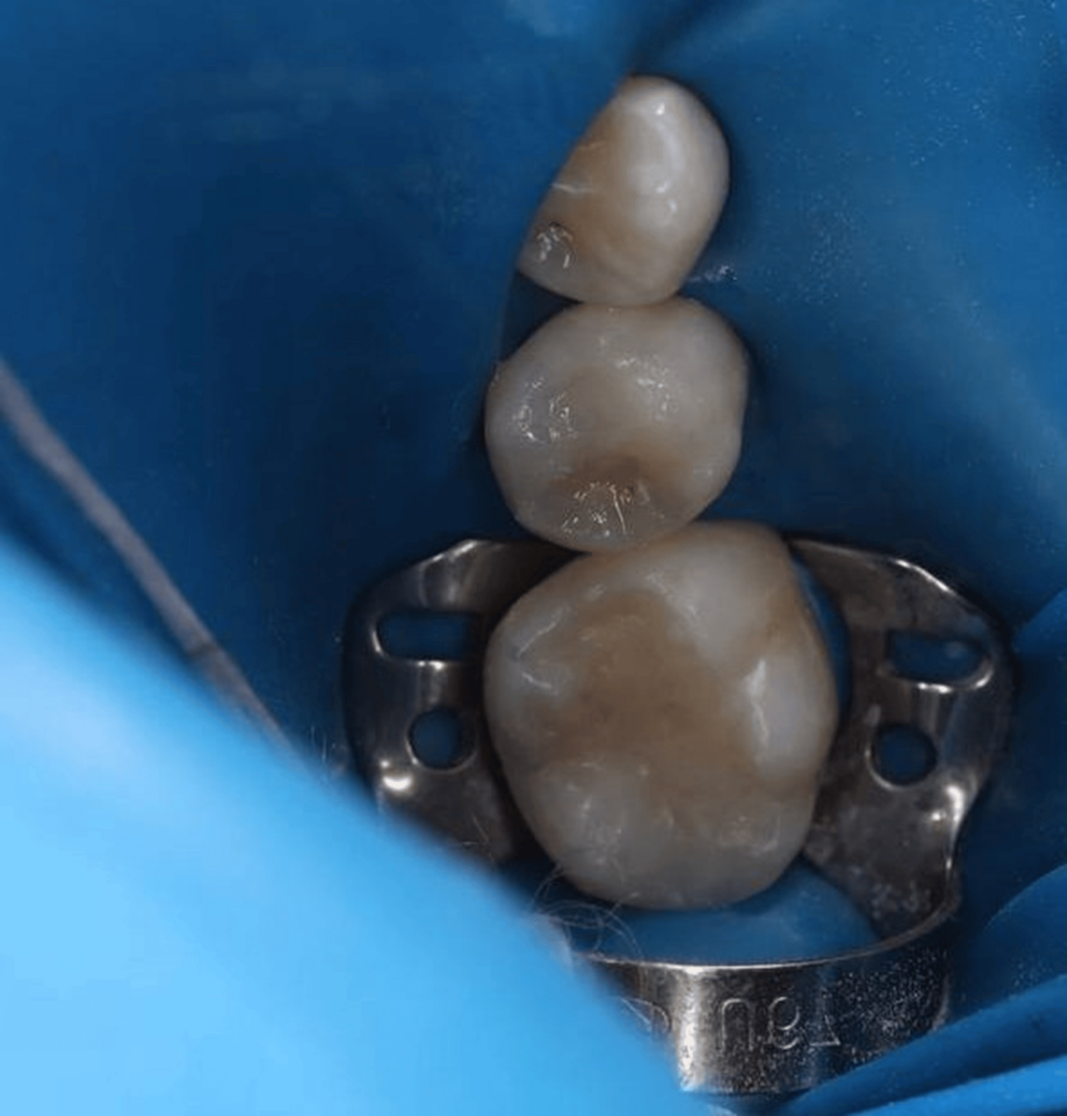
Completed composite restoration demonstrating proper anatomic contour and surface adaptation.

**Figure 6:**
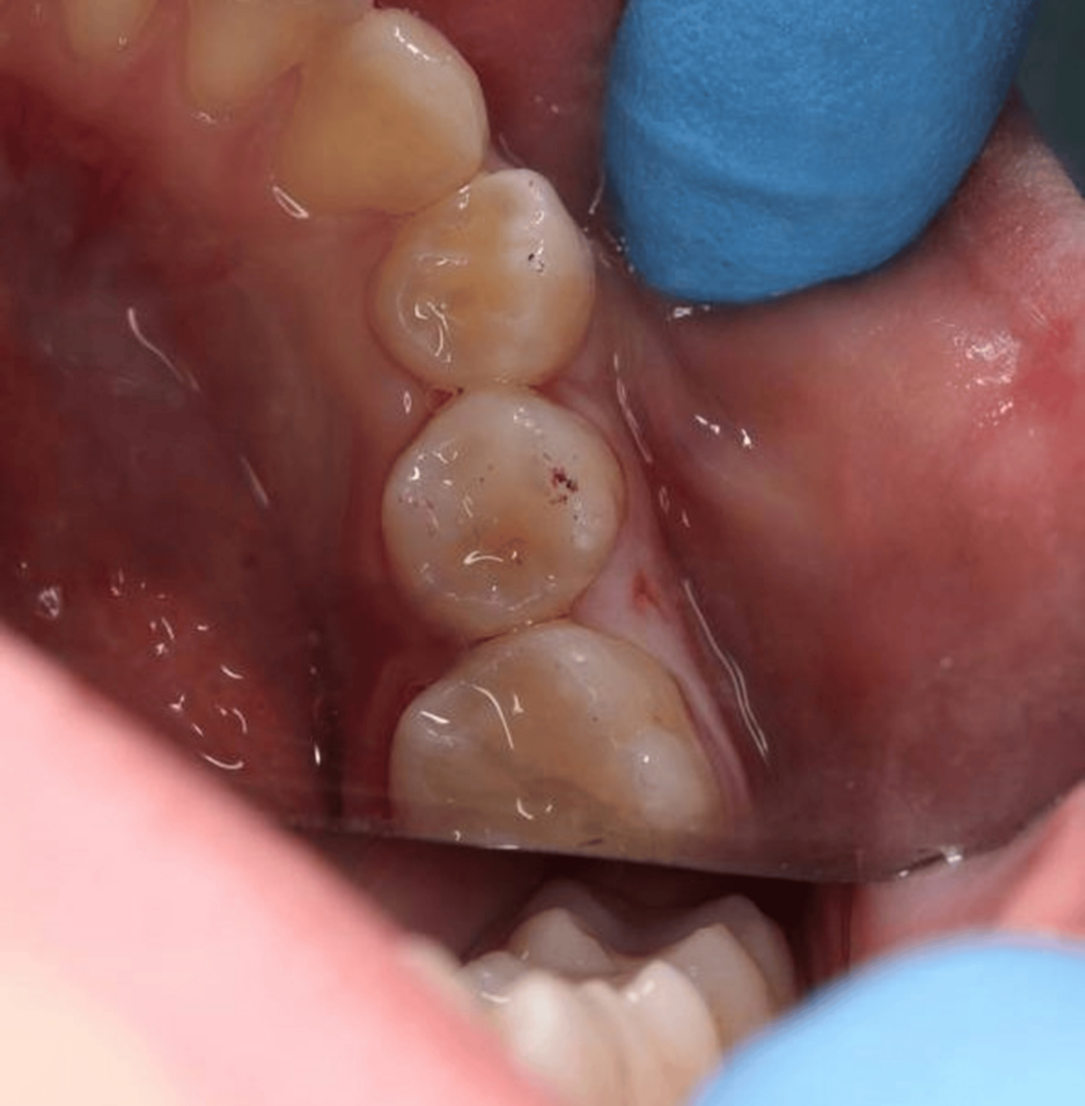
Final restoration after occlusal adjustment and polishing, demonstrating a smooth surface finish and correct occlusal contacts.

The cavity was etched with 37% phosphoric acid for 30 seconds, followed by application of Kerr OptiBond Extra Universal adhesive according to the manufacturer's instructions. The cavity was then restored with Estelite Sigma Quick composite resin (shade A1), as illustrated in Figure 5. Occlusion was checked and adjusted as needed, and the restoration was polished to a smooth, glossy finish (Figure 6).

**Figure 7:**
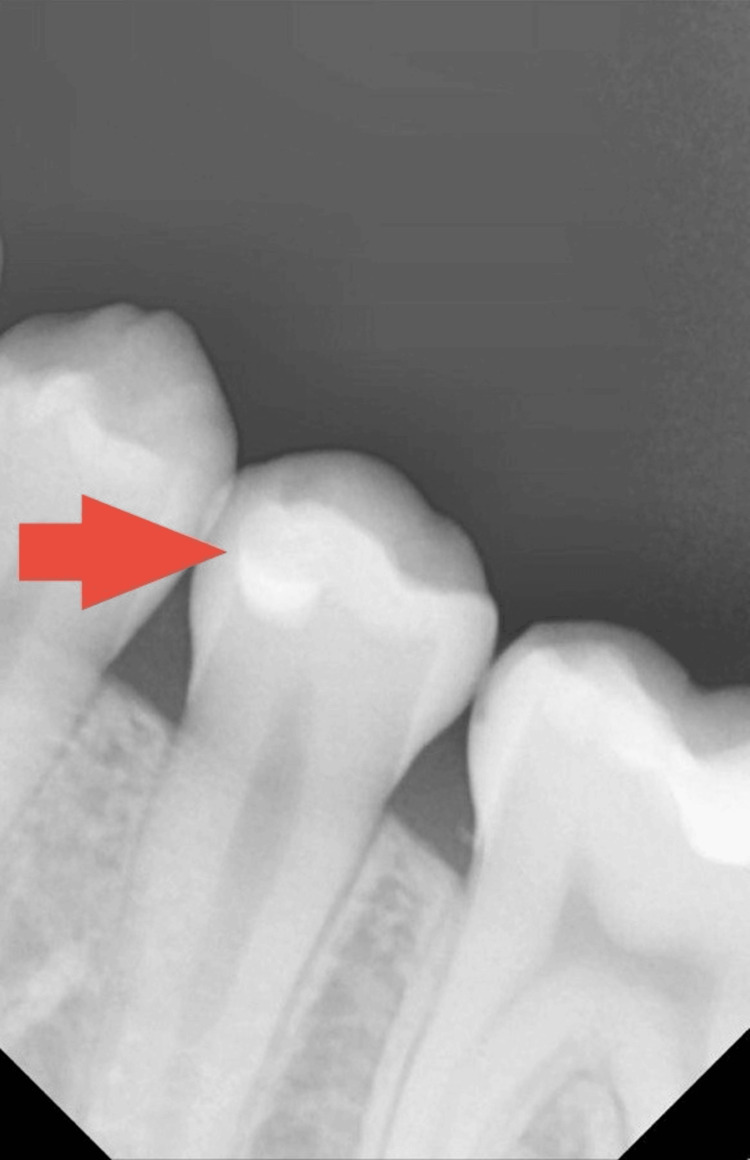
Postoperative periapical radiograph showing complete removal of carious tissue, adequate marginal seal, and proper restoration adaptation on tooth #20.

A postoperative periapical radiograph (Figure 7) confirmed complete removal of carious tissue and proper marginal adaptation of the restoration. The patient tolerated the procedure well without any discomfort or complications.

A comprehensive intraoral examination, photo documentation, and radiographic assessment were performed to ensure treatment quality. The procedure followed standard conservative restorative principles [4], and rubber dam isolation provided optimal moisture control and patient safety. A follow-up appointment was scheduled to evaluate the integrity of the restoration and the long-term vitality of the tooth.

The hidden carious lesion in tooth #20 was successfully treated without pulp involvement. The minimally invasive approach allowed for the preservation of healthy tooth structure, reduced treatment time, and prevented the need for endodontic therapy. The final restoration was functional, esthetic, and durable, while tooth vitality was fully preserved.

## Discussion

This case highlights the critical role of routine radiographic examination in identifying early carious lesions that may remain undetectable during clinical inspection. Despite the absence of symptoms and clinically visible changes, the periapical radiograph revealed a hidden occlusal carious lesion on tooth #20. Such cases emphasize that visual and tactile examination alone may be insufficient, particularly in the posterior region, where pits and fissures can mask subsurface demineralization.

The ADA and the U.S. Food and Drug Administration (FDA) recommend individualized radiographic assessment based on caries risk, rather than a fixed interval for all patients [5]. In children and adolescents, where the rate of caries progression is higher, routine radiographs at appropriate intervals serve as an essential adjunct to clinical examination for early detection and prevention of extensive decay. This case clearly demonstrates the practical application of these guidelines, as the lesion would likely have remained undiagnosed until significant structural compromise or pulpal involvement occurred.

Radiographically detected hidden lesions, especially in young permanent teeth, often originate in deep occlusal fissures or under an apparently intact enamel surface [6]. Without early detection, such lesions can progress rapidly, necessitating endodontic intervention or even extraction. In this patient, a timely radiographic diagnosis enabled a preventive, conservative approach that successfully preserved pulp vitality. The minimally invasive management reflected the modern paradigm of caries treatment, focusing on early detection, selective removal of infected dentin, and maximal preservation of healthy tissue.

The use of rubber dam isolation [7], selective caries excavation, and adhesive restorative techniques followed evidence-based standards for ensuring long-term success. Proper isolation minimizes contamination and improves the bond strength of resin composites. The use of an etch-and-bond system (OptiBond Extra Universal) [8] combined with a nanohybrid composite (Estelite Sigma Quick) [9] provided optimal marginal adaptation, esthetics, and durability. Additionally, postoperative radiographic confirmation ensured that the restoration was well-adapted and free from overhangs or voids, both of which could predispose to secondary caries [10].

The decision to use a conservative approach, rather than extensive preparation or indirect restoration, aligns with the concept of "minimally invasive dentistry" (MID) [11]. MID promotes preservation of natural tooth structure, improved longevity, and patient comfort, while maintaining biological, mechanical, and esthetic integrity. In pediatric and adolescent patients, such as in this case, maintaining pulp vitality is particularly important for continued root development and long-term tooth survival.

This case also underscores the psychological and educational importance of patient and guardian communication. Explaining the rationale for radiographs and conservative treatment not only increases cooperation but also helps dispel the misconception that X-rays are unnecessary in asymptomatic patients. Effective communication contributes to long-term adherence to preventive care, regular check-ups, and overall oral health awareness.

In summary, this case illustrates how a routine radiographic examination can transform an apparently routine dental visit into an opportunity for early intervention and prevention of complex pathology.

## Conclusions

This case underscores the essential role of routine radiographic examination in contemporary dental practice. Even in asymptomatic patients with clinically intact teeth, hidden carious lesions can progress silently beneath the enamel surface. Early radiographic detection allows clinicians to intervene at the optimal stage, preventing pulp involvement, minimizing tooth structure loss, and avoiding complex restorative or endodontic procedures.

The successful management of tooth #20 demonstrates that adherence to minimally invasive, evidence-based treatment principles ensures both biological and functional success. Careful diagnosis, precise operative technique, and the use of modern adhesive materials under proper isolation result in restorations that are durable, esthetic, and preserve tooth vitality. Ultimately, this case highlights the importance of comprehensive, individualized care, where clinical examination, radiographic assessment, and patient education work together to promote long-term oral health and trust between the dentist, patient, and family.
